# Private speech: similarities between a large language model and children

**DOI:** 10.3389/frai.2026.1691074

**Published:** 2026-01-29

**Authors:** Zhiyu Liang, Leon On Tay, Simon Dennis

**Affiliations:** 1 Melbourne School of Psychological Sciences, The University of Melbourne, Melbourne, VIC, Australia; 2 Intelligent Silicon Pty Ltd., Melbourne, VIC, Australia

**Keywords:** consciousness, developmental psychology, inner speech, large language model, private speech

## Abstract

This study investigates the capability of a non-reasoning large language model (GPT-4o) to generate private speech and evaluates its similarity to human private speech. We placed the model in a simulated solitary block-construction scenario via textual prompts, eliciting and classifying its self-directed utterances using an established semantic framework for categorizing private speech in children. The distribution of these categories was compared to two human benchmarks: a classic block-construction study and a more recent experiment employing a similar task setting. Analysis using scatter plots and Pearson correlation coefficients revealed a striking pattern: GPT-4o’s semantic profile showed negligible similarity to the classic benchmark (*r* = 0.01) but very strong similarity to the recent benchmark (*r* = 0.93). This discrepancy is interpreted as stemming from differences in task nature, namely goal-directed, scaffolded task versus self-determined, unscaffolded play, which exert a stronger influence on speech content than experimental subject difference between GPT-4o and children. In an exploratory serial recall study, we tasked GPT-3.5-Turbo-instruct and observed incidental private speech, indicating that the phenomenon extends across contexts. This provides an avenue for investigating LLM replication of private speech and, potentially, computational consciousness.

## 1 Private speech in human research

Private speech refers to the phenomenon where individuals, particularly children, talk aloud to themselves during activities. It is distinct from social speech, as it is not directed at others. Private speech is considered a transitional form of communication that bridges social speech and inner speech, the latter being the internalized, silent form of self-dialogue (Vygotsky, 1962; Winsler and Naglieri, 2003).

### 1.1 Theories of private speech

Although the phenomenon of private speech was first studied by Piaget (1955), who interpreted it as “egocentric speech” reflecting children’s cognitive inability to adopt others’ perspectives, it was Vygotsky’s (1962) theory in *Thought and Language* that gained prominence. Vygotsky contested Piaget’s view, arguing that such speech is not a deficit but a vital transitional stage toward self-regulation, ultimately evolving into inner speech.

Central to Vygotsky’s framework was the idea that private speech arises from early social interactions, particularly during cognitively challenging tasks within the Zone of Proximal Development, defined as the range of tasks a child can accomplish with guidance from more knowledgeable others. Through collaborative problem-solving, children internalize language from social exchanges, transforming it into self-directed speech. This process enables a shift from other-regulation to self-regulation, with private speech acting as a tool for thought. Over time, it becomes internalized as silent inner speech, serving as the foundation for higher cognitive functions.

Inner speech, or covert/silent speech, or inner verbal thought, refers to the silent, internal use of language in thinking (Alderson-Day and Fernyhough, 2015). According to Vygotsky’s sociocultural theory, young children’s private speech initially demonstrates explicit verbal expression and interpersonal communicative qualities; however, it gradually becomes abbreviated and internalized, shedding phonetic articulation and syntactic complexity until fully transitioning into silent inner speech (Vygotsky, 1987). Thus, Vygotsky’s theory holds that private speech eventually “goes underground” and transforms into the abbreviated, covert inner speech used by older children and adults (Vygotsky, 1987). Empirical developmental studies have provided support for this internalization process by showing gradual phonetic reduction in children’s private speech (Berk, 2014) and its functional link to cognitive development (Winsler et al., 2009).

Subsequent research has expanded on Vygotsky’s ideas, exploring the developmental trajectory, functions, and methodologies for studying private speech.

### 1.2 Developmental trajectories of private speech

Vygotsky’s initial theory proposed that private speech follows a curvilinear, inverted U-shaped trajectory across childhood: overt self-talk becomes increasingly frequent, peaks during the preschool years, and then declines in early elementary school as it transitions to whispered speech, inaudible muttering, and eventually silent inner verbal thought. Over time, research has provided partial support for this broad developmental trend. While there is strong evidence that private speech shifts from overt, externalized forms to more internalized modes as children age, the hypothesis that specific ages rigidly mark the emergence or disappearance of private speech lacks robust empirical backing (Berk, 2014).

Overall, two developmental trajectories are supported by research. At a broader developmental level, private speech follows a general trajectory: overt self-talk is most frequent during early childhood, peaking in the preschool years, and gradually becoming more internalized and less outwardly observable by around age 7 or 8 (Behrend et al., 1989; Kohlberg et al., 1968). However, a smaller-scale, immediate pattern also exists within individuals of any age when they tackle cognitively demanding tasks. Here, overt private speech surges during initial struggles with the task and diminishes as the person gains proficiency over time or through repeated practice (Duncan and Pratt, 1997; Duncan and Cheyne, 2001).

### 1.3 Functions of private speech

Private speech plays a crucial role in task regulation and problem-solving by helping children plan and execute complex actions (Berk, 1992). When engaged in tasks like building a block tower, children verbalize each step (e.g., “I’ll put the green one here, then the red one on top”), which reinforces memory and guides behavior (Alderson-Day and Fernyhough, 2015; Winsler et al., 2009). This self-monitoring mechanism aids in error correction, as children recognize and adjust mistakes aloud (e.g., “That looks crooked, I need to fix it”), allowing for immediate feedback and strategic adjustments (Berk, 1992). By vocalizing instructions or repeating key details (e.g., “First, three blocks go here…one, two, three”), it also enhances short-term recall through verbal rehearsal (Winsler et al., 2009).

Beyond cognitive regulation, private speech serves an emotional and motivational function (Vygotsky, 1962). Children often use self-encouragement (e.g., “I can do this!”) to maintain focus and confidence in challenging situations (Berk and Spuhl, 1995). Verbalizing anxieties (e.g., “I’m nervous about this part”) can also help manage stress and sustain engagement in problem-solving (Winsler et al., 2003). Studies show that children who frequently use private speech tend to persist longer and perform better on difficult tasks (Berk and Winsler, 1995; Fernyhough and Fradley, 2004).

### 1.4 Research methods of private speech

Private speech is commonly researched using observational methods, including naturalistic observation and laboratory-based observation. These approaches allow for systematic analysis of how and when private speech emerges in real-world settings and controlled conditions.

Naturalistic observation involves studying children in familiar environments, such as homes, classrooms, or playgrounds, without interference from researchers (Winsler et al., 2009). This method provides ecologically valid data, capturing spontaneous private speech during everyday activities like playing, problem-solving, or completing schoolwork. For instance, researchers might observe children talking to themselves while building with blocks or solving puzzles, analyzing how speech guides their actions and adapts to task complexity (Berk, 1992). A key advantage is that it reflects authentic behavior, but a limitation is the lack of experimental control, making it difficult to establish causality.

In contrast, laboratory observation involves structured tasks in controlled settings, allowing researchers to manipulate variables and examine private speech under specific conditions (Berk, 1992). Tasks such as puzzle-solving or serial recall memory exercises (Elliott et al., 2021) are able to elicit private speech, enabling systematic comparison across different age groups or cognitive abilities. This method enhances reliability and reproducibility.

## 2 Large language models and private speech

Transformer-based large language models (LLMs), as introduced by Bahdanau et al. (2014) and further developed by Vaswani et al. (2017), have demonstrated significant prowess in mimicking cognitive functions traditionally attributed to specialized cognitive frameworks (Piantadosi, 2023). For example, research by Webb et al. (2023) highlights that models like GPT-3 exhibit a capability to spontaneously generate solutions for a wide array of analogy challenges without prior specific training. Despite some criticisms aimed at the transformer architecture’s proficiency in handling complex cognitive tasks (Han et al., 2022; Mahowald et al., 2023; Binz and Schulz, 2023; Chomsky et al., 2023), these concerns have largely been mitigated as the models have grown in size and the datasets used for training have become more comprehensive (Han et al., 2024).

Building on the literature on the cognitive capabilities of LLMs, which have been shown to simulate human-like functions such as personality traits (Jiang et al., 2024), theory of mind reasoning (Kosinski, 2024) and self-directed problem-solving (Bubeck et al., 2023), we asked a further question: to what extent can LLMs produce private speech? And if LLMs can produce private speech, how similar is it to human private speech? Our study, therefore, investigates whether an LLM spontaneously produces private-speech-like utterances when placed in an analogue of the classic laboratory paradigm and how closely the form and frequency of any such output match the patterns documented in human participants.

### 2.1 Reasoning traces and private speech

A reasoning model (reasoning LLM) refers to an LLM explicitly trained to solve complex tasks by mimicking structured, logical problem-solving processes. Unlike non-reasoning models that generate answers directly, reasoning models produce intermediate “reasoning traces.” They are step-by-step logical sequences similar to a human’s internal monologue when tackling challenges. These traces act as a scaffold for systematic thinking, enabling the model to decompose problems, test hypotheses, and refine conclusions before finalizing a response. This mechanism parallels human inner speech to some extent. Human inner speech and the reasoning traces of LLMs both manifest, at least superficially, as language-mediated cognitive processes. Inner speech is commonly employed by humans for mental operations such as silently narrating steps, posing questions, and simulating dialogues. Similarly, reasoning models demonstrate problem-solving capacity through linguistic mediation, where reasoning traces (e.g., chain-of-thought outputs) express the sequential processes of models generating outputs via language-based representations.

Efforts have been made to investigate LLM and inner speech. Most works, similar to the line of research promoting the reasoning capabilities (e.g., chain-of-thought prompting) in LLMs, have been trying to configure inner speech capability in language models or artificial agents in order to perform specific tasks to detect improvement in performance. For example, Pipitone and Chella (2021) designed an inner speech cognitive architecture which allows robots to verbally label the perceived entities and talk to themselves. Benefiting from the conceptual reasoning of inner speech, such a robot passed the mirror test. Similarly, Huang et al. (2022) developed an inner monologue system by providing embodied environment feedback to an LLM, which they applied to assist a robotic agent in performing tasks. Their results showed that the inner monologue-assisted robot achieved a higher success rate compared to both traditional methods and an LLM without the embodied feedback. Additionally, their findings demonstrate that inner monologue enables emergent capabilities absent explicit prompting, including self-initiated goal revision during plan infeasibility and continuous adaptation to human instructions.

However, not much work was done on investigating the spontaneous capability in LLM inner speech. Philosophical investigations (e.g., Mann and Gregory, 2024) provide mixed evidence regarding the existence of inner speech in LLMs based on a Turing-like approach. In their study, the authors tested text-davinci-003 through dialogue tasks (direct queries, final-word extraction, and rhyme detection). While the model explicitly claimed to possess inner speech and succeeded in partial tasks, its inconsistent performance on non-word rhyme tasks revealed contradictory rationales. Mann and Gregory argue that LLMs operate as statistical next-word predictors, rendering observed behaviors insufficient to attribute inner speech. Drawing on developmental psychology, our work investigates an LLM’s spontaneous capabilities in generating private speech. We aimed to adapt experimental designs from this field and compare the LLM’s performance with human benchmarks.

### 2.2 Reasoning model versus non-reasoning model

To investigate spontaneous private speech–like behavior in language models, we deliberately chose to employ non-reasoning. The training corpus of the reasoning models is augmented with reinforcement-learning methods that explicitly optimize step-by-step reasoning. Hence, using non-reasoning models without this additional augmentation provides a baseline for assessing whether self-directed utterances emerge organically from the model’s learned textual patterns, rather than from explicit prompting and training. By contrast, a reasoning-enabled architecture is trained to maintain and update hidden traces, thereby effectively modeling inner thoughts, such as planning statements and self-evaluations (Wei et al., 2022; Bubeck et al., 2023). Using a non-reasoning model thus avoids artificially boosting self-regulatory content and ensures that any private-speech phenomena we observe truly arise from a model’s default generation process.

### 2.3 Testing non-reasoning large language model with private speech task

Our goal is to determine whether an LLM, placed in a private speech task context, exhibits analogous self-directed speech patterns. Winsler et al. (2003) developed a 10-category classification system that provides a granular, semantic classification, distinguishing categories such as self-guiding directives, task-relevant descriptions, and motivational statements. This framework is ideal for analyzing LLM-generated private speech as it allows for comparisons with established human data and aligns with private speech tasks.

Prior research by Winsler et al. (2003) found that human children produce private speech, which they classified into 10 categories, namely, Exclamations, Descriptions of Task/Environment, Nonwords, Descriptions of self, Evaluative/Motivational statements, Plans/Hypothetical Reasoning, Commands to Self, Questions/Answers, Transitional Statements, and Other utterances. Exclamations capture brief affective bursts (e.g., “oh,” “oops”). Descriptions of Task/Environment note properties of the materials or context (e.g., “this piece is blue”). Nonwords are vocalizations without lexical content (e.g., sound effects, humming). Descriptions of Self are statements about one’s state or behavior (e.g., “I am stuck”). Evaluative/Motivational Statements include self-praise, critique, or effort statements (e.g., “this is hard, but I can do it”). Plans/Hypothetical Reasoning cover future-oriented or conditional planning (e.g., “first I sort, then I build”). Commands to Self are imperatives that guide one’s actions (e.g., “put this here”). Questions/Answers are queries posed to oneself, optionally followed by an answer (e.g., “where does this go… here”). Transitional Statements signal shifts between steps or phases (e.g., “okay, next”). Other utterances encompass content not captured in the different categories.

To identify a contemporary and culturally distinct replication of Winsler’s task, we systematically screened the citing literature and identified a recent study by Uçar and Sofu (2021). Their work applied Winsler’s categorization system and construction task in a Turkish context under a free-play setting without scaffolding, effectively replicating the paradigm under more naturalistic conditions. By comparing against both the original paradigm and its contemporary, similar study, we can examine whether the distribution of semantic categories in LLMs resembles data observed in humans across cultural contexts and in a more up-to-date developmental cohort.

Testing an LLM within this established human experimental framework allows us to explore whether artificial models, like children, employ language in ways that parallel private speech, thus offering new insights into both LLM-generated language and the cognitive underpinnings of self-directed speech.

## 3 Method

To examine whether a non-reasoning LLM can generate private speech, we adapted a classic developmental psychology paradigm for use with an LLM. The core of our approach was to take an established experimental task and implement it through carefully designed textual prompts.

The following sections detail the data sources, experimental stimuli, procedural setup, and analytical methods.

### 3.1 Data and experimental stimuli

Our study utilized two primary sources of data: (1) two human benchmark datasets from established developmental psychology research, and (2) a novel dataset of LLM-generated utterances collected through our experimental procedure.

#### 3.1.1 Human benchmark data

We used the private speech data from Winsler et al. (2003) as our first human baseline for comparative analysis. To derive the proportional distribution for the 10 semantic categories, we extracted the mean number of utterances per category for the block-construction task at Time 1 (T1) from their Table 1. We then summed the mean utterances across all 10 categories to obtain a total and calculated the proportion of each category by dividing its corresponding mean number by this total. This derived proportional distribution is based on data from *N* = 32 children (sixteen 3-year-olds and sixteen 4-year-olds).

We also used the private speech data from Uçar and Sofu (2021) as the second human baseline for comparative analysis. While they used children aged 3–5 years old, we only used their data of children aged 3–4 years, as this age range represents the peak of overt private speech before it begins to internalize. Classic and subsequent studies show that younger preschoolers produce substantially more audible self-directed speech than older children (Piaget, 1955; Klein, 1964; Kohlberg et al., 1968), making this developmental window ideal for observing private speech in its most externalized form. To derive the proportional distribution of the 10 semantic categories, we extracted the mean values for each category across the two age groups from their Table 3. For each category, we summed its mean numbers across the two age groups. The total was calculated by summing the mean numbers of all 10 categories, after merging “Questions/Answers of the Imaginary Characters” and “Questions/Answers to the Self” into a single “Questions/Answers” category, across the three-year-olds and four-year-olds. The proportion of each semantic category was then calculated by dividing its aggregated mean by this total. This derived proportional distribution is based on data from *N* = 18 children (eight 3-year-olds and ten 4-year-olds).

#### 3.1.2 LLM-generated data

##### 3.1.2.1 Input prompts (stimuli)

The core input to the LLM consisted of a structured system prompt designed to simulate a solitary play scenario. The prompt stated “You are a three/four-year-old child in a room that contains playing blocks on the floor. You are the only person in the room; there is no one else here to talk to.” The three/four placeholder was varied across trials to match the age distribution in the human study.

##### 3.1.2.2 Output corpus

The model GPT-4o’s text-based responses were collected via the OpenAI API (Hurst et al., 2024). We segmented the output to isolate utterances from descriptions of action (e.g., *Walks over to the blocks and starts picking them up one by one*), resulting in a final corpus of 509 LLM-generated utterances for analysis (e.g., “Ooo, blocky!”). The number of utterances per trial ranged from 22 to 94 (Median = 65.5). This dataset of annotated LLM utterances is publicly available at: https://osf.io/t3us2/.

### 3.2 Experimental task and procedure

The selection of the Block-construction task (Winsler et al., 2003) was guided by an evaluation of the feasibility and suitability of tasks traditionally used to study human private speech within the unique constraints of LLMs as experimental subjects. While multimodal LLMs (e.g., GPT-4o) possess nascent vision comprehension capabilities, pilot testing revealed significant practical limitations. Specifically, attempts to adapt vision-comprehension tasks like the sequencing task (Frauenglass and Diaz, 1985) encountered substantial challenges: (1) Current multimodal APIs presented technical hurdles for seamless image integration and processing within our experimental pipeline, and (2) more critically, preliminary testing (via the UI) revealed that GPT-4o’s understanding and execution of visual reasoning tasks were insufficiently reliable to meet our research needs. The construction task could be adapted and, therefore, mediated solely through textual instruction, offering a highly feasible and controlled paradigm. It allows us to present a scenario that inherently elicits self-directed verbalisation within a non-social context (i.e., the LLM is prompted as if alone, focusing solely on the task), aligns well with the textual nature of LLM output, and, crucially, provides a direct benchmark against established human data for comparative analysis. We therefore chose the block-construction task, which allows us to compare the performance of LLMs with humans by setting the scenario with prompts.

As detailed in Section 3.1.2, the GPT-4o model was placed in the simulated solitary play scenario using the designed prompt. To emulate a cumulative monologue, each independent trial involved 20 sequential model responses, with the model’s prior output appended to its message history to provide context for subsequent replies. The model’s token output was constrained to 60 tokens per response to ensure brevity.

We conducted a total of eight independent trials (context was reset between trials), with four trials per age condition (three-year-old and four-year-old). No user inputs were provided after the initial prompt.

### 3.3 Speech coding and classification

We classified all LLM utterances into the 10 semantic categories defined by Winsler et al. (2003): Descriptions of the Environment/Task, Plans/Hypothetical Reasoning, Evaluative /Motivational Statements, Questions/Answers, Nonwords, Exclamations, Descriptions of the Self, Commands to the Self, Transitional Statements, and Other utterances.

Two researchers independently classified all 509 speech utterances. The interrater reliability, measured by Cohen’s kappa, was 0.91 across the eight trials, which is considered almost perfect agreement (Landis and Koch, 1977). For the final analysis, we used the average of the two researchers’ category distributions as the resulting distribution for the LLM.

### 3.4 Data analysis

Rather than aiming to test for statistically identical proportions across categories, our analysis sought to evaluate the similarity in overall semantic profiles of private speech between the LLM and human children. Accordingly, we utilized scatter plots and Pearson correlation coefficients to analyze three comparison pairs: the LLM versus Winsler et al. (2003), the LLM versus Uçar and Sofu (2021), and Winsler et al. (2003) versus Uçar and Sofu (2021).

## 4 Result

We screened all 509 LLM utterances against Winsler et al.’s (2003) criteria, with every utterance classified into one of their 10 private-speech categories. To assess whether LLMs are capable of generating private speech, we analyzed the utterances produced by the model. To decide the degree of similarity of the semantic profile of private speech generated by the model to those of human benchmarks, we compared the distribution of utterance categories among the three data sources.

### 4.1 Capacity for generating private speech

GPT-4o was found to generate speech that meets the criteria for private speech, as the utterances were not directed at another subject except for itself and often consisted of self-directed descriptions of movement and thought processes. The model demonstrated the ability to produce speech aligned with internal reasoning, self-regulation, and task-related descriptions, indicating that LLMs can effectively simulate private speech.

### 4.2 LLM-human comparisons and benchmarks comparison

**Figure 1:**
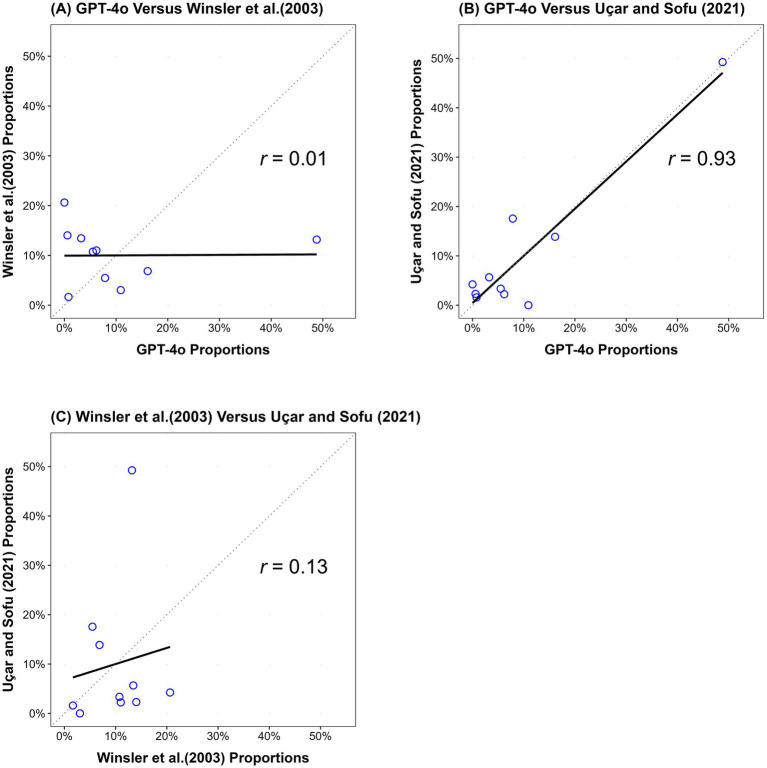
Scatter plots of LLM-human comparisons. **(A)** Comparison of category proportions between GPT-4o and Winsler et al. (2003), across 10 categories. **(B)** Comparison of category proportions between GPT-4o and Uçar and Sofu (2021), across 10 categories. **(C)** Comparison of category proportions between Winsler et al. (2003) and Uçar and Sofu (2021), across 10 categories. Each dot represents one of the 10 categories.

Figure 1 presents a comparison among three data sources, GPT-4o, Winsler et al. (2003), and Uçar and Sofu (2021) via scatter plots with fitted correlation lines to illustrate their linear relationships.

Plot A reveals negligible similarity between the semantic profiles of GPT-4o and Winsler et al. (2003), with a correlation coefficient of *r* = 0.01. This indicates a very weak correlation, consistent with a negligible effect size according to conventional guidelines (Cohen, 1988).

Plot B demonstrates a near-perfect similarity between the semantic profiles of GPT-4o and Uçar and Sofu (2021), with a correlation coefficient of *r* = 0.93. This represents an exceptionally strong correlation (Cohen, 1988).

To meaningfully interpret the correlation between the LLM and human benchmarks, it is essential to consider the baseline level of similarity observed among existing human studies. Plot C shows a slight similarity between the semantic profiles of Winsler et al. (2003) and Uçar and Sofu (2021), with a correlation coefficient of *r* = 0.13. This suggests a weak correlation (Cohen, 1988).

**Figure 2:**
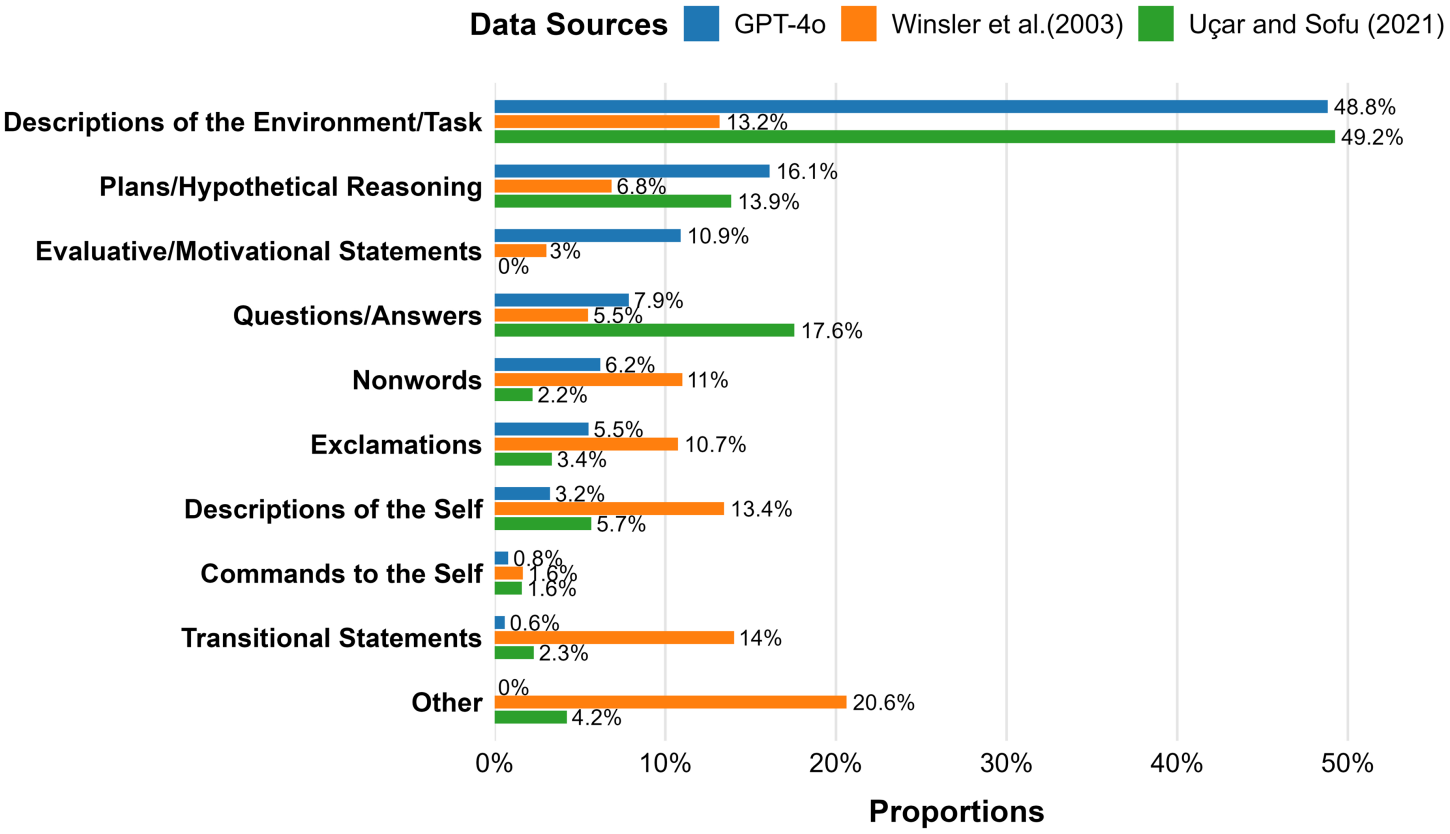
Distribution of category proportions across the three data sources. Percentages may not sum to 100% due to rounding.

As shown in Figure 2, when comparing category proportions across the three data sources, GPT-4o demonstrated a pattern that was highly aligned with Uçar and Sofu (2021) but diverged from Winsler et al. (2003). Specifically, GPT-4o and Uçar and Sofu (2021) both showed substantially elevated proportions of Descriptions of the Environment/Task relative to Winsler et al. (2003). GPT-4o unlike Uçar and Sofu (2021), overproduced Evaluative/Motivational statements compared to Winsler et al. (2003). GPT-4o underrepresented categories that were more prominent in Winsler et al. (2003), including Descriptions of the Self, Transitional Statements, and Other utterances. Compared to Uçar and Sofu (2021), GPT-4o overproduced Evaluative/Motivational Statements, and underproduced Questions/Answers and the Other category. Uçar and Sofu (2021), compared to Winsler et al. (2003), similarly overproduced Descriptions of the Environment/Task and Questions/Answers, but underproduced Transitional Statements, Exclamations, Nonwords, Descriptions of the Self, and Other utterances.

## 5 Discussion

Overall, we aimed to investigate the capacity of a non-reasoning LLM to generate private speech and the extent to which private speech is similar to that of humans in terms of the category distribution. Congruent with prior investigations of LLM capabilities (Serapio-García et al., 2023; Kosinski, 2024; Betley et al., 2025), we found that GPT-4o is capable of generating speech that is not addressed to anyone, which adheres to the definition of private speech. Our experiments demonstrate that GPT-4o, trained on human corpora, exhibits characteristics of human-like private speech patterns. We found that the proportions of categories for private speech generated by GPT-4o showed negligible similarity from the proportions of Winsler et al. (2003) but strong similarity to the proportions of Uçar and Sofu (2021). For context, the proportions of Uçar and Sofu (2021) and Winsler et al. (2003) are weakly related, indicating that our findings are likely due to task nature differences rather than general equivalence to human private speech.

When examining the scatterplots of category proportions, certain categories showed notable descriptive deviations, namely Descriptions of the Environment/Task, Evaluative/Motivational Statements, Questions/Answers, Transitional Statements and Other.

Notably, a striking disparity exists in category prevalence between GPT-4o and Winsler et al. (2003): Descriptions of the Environment/Task (e.g. “so many toys!”) dominate GPT-4o outputs, constituting 48.8% of all generated content. The significant prevalence of environmental and task-related descriptions in GPT-4o-generated private speech may stem from their substantial presence in training corpora. When writing for absent readers, humans must explicitly describe the observable setting, ongoing tasks, their progress, and resulting environmental alterations, necessitating extensive descriptive passages. GPT-4o internalized this characteristic textual feature during training, consequently replicating the emphasis on environment and task descriptions in its private speech output.

GPT-4o did not produce any utterances in the Other category. The Other category refers to utterances that do not belong in the nine other categories (Winsler et al., 2003). GPT-4o underproduced compared to children in both benchmarks (0% vs. 20.6% from Winsler et al. (2003) and 4.2% from Uçar and Sofu (2021). The absence of output sorted into the other category provides greater evidence for the effect of its instruct training on the output. For human children, an example of output in that category could be a non-task-relevant utterance. However, due to GPT’s instruct training, the model is tuned to generate output related to the prompt, hence it is unlikely to generate off-topic utterances.

The prevalence of Evaluative/Motivational Statements in GPT-4o-generated private speech may stem from its training corpus. The training corpus of GPT-4o comprises 60% Common Crawl (webcrawl data), 16% books, 3% Wikipedia and 21% other web text (Brown et al., 2020), hence GPT-4o could be more frequently exposed to explicit expressions of evaluation and encouragement due to their use by authors to structure narratives, or maintain reader engagement. Conversely, the brief private speech utterances muttered during tasks by children would be under-represented in the corpus (the source being transcripts of experiments). GPT-4o’s instruction tuning together with reinforcement learning from human feedback (RLHF) further encourages supportive, confidence-building phrasing (Ouyang et al., 2022). Instruction tuning refers to training the LLMs on exemplars demonstrating how the LLMs should respond (i.e., helpful, polite, and explicitly supportive). RLHF refers to post-training refinement of LLM behavior based on human preferences, ensuring that it is helpful, harmless, and honest (Ouyang et al., 2022). These factors plausibly amplify this category.

GPT-4o underproduced Transitional Statements compared to Winsler et al. (2003). However, it produced a similar proportion compared to Uçar and Sofu (2021). The mechanism behind this phenomenon could plausibly be attributable to differences in task nature across our study Winsler et al. (2003) and Uçar and Sofu (2021), specifically the contrast between open-ended and goal-directed scenarios.

The observed variation in similarity when comparing GPT-4o output to the two distinct human benchmarks may be better explained by differences in task nature between the benchmarks themselves, rather than by the subject (GPT-4o vs. children). Specifically, it appears to stem from whether the setting is structured and scaffolded, or open and self-determined. Both Uçar and Sofu’s (2021) work and our work employed a play-based context with minimal scaffolding and no prescribed goal, using simple scenarios (e.g., freely arranged items) to elicit spontaneous private speech. This shared self-determining nature aligns with literature suggesting that private speech during open-ended activities reflects child-selected topics and self-defined tasks (Krafft and Berk, 1998), which likely contributes to the higher similarity in semantic profiles between these two. In contrast, Winsler’s paradigm involved a clear, scaffolded goal (e.g., reproducing a specific model), which constrains self-determination and orients speech toward instruction-following and recall, resulting in a differing semantic profile. Therefore, the task nature seems to exert a stronger influence on private speech content than the subject difference between language models and children.

### 5.1 Evidence for incidental private speech by LLMs

Here, we distinguish incidental private speech, defined as utterances that emerge during tasks not designed to elicit self-talk and without any instruction to think aloud, from spontaneous private speech, defined as utterances produced when the model is placed in an open-ended context that affords self-talk but does not require specific content.

A critical limitation arises from our methodological framework: all model outputs were elicited through prompts, though the prompts were designed to avoid requiring direct responses. Such an issue might be called the prompt paradox, whereby providing prompts that direct answers, such as through chain-of-thought prompting, results in the output being compliant to the prompt rather than true self-regulation (Wei et al., 2022). The construction-task data, for example, rely on a child-play prompt that implicitly licenses narrative continuations; hence, critics can plausibly argue that the utterances merely echo child-story templates present in the training corpus (Bubeck et al., 2023). However, can LLMs autonomously generate private speech without explicit prompting, mirroring the spontaneous private speech observed in human cognitive development?

To address this, we propose adapting the analysis methodology from developmental psychology research on private speech. In human studies, children’s private speech generated during task performance (e.g., block-construction activities) is systematically analyzed, independent of the task performance. By analogy, we seek to investigate whether LLMs can generate incidental private speech during task performance, that is, self-directed verbalisations distinct from their prompted outputs, without any attempt to prompt private speech.

To investigate this issue, we ran an exploratory serial-recall study focused on manipulating memory load. We tested GPT-3.5-Turbo-instruct. This model was used as other more advanced models exhibited the ceiling effect. The model received a single prompt, “Now recall the list in order,” for lists of 100, 200, and 300 items. No mention was made of strategies or emotional responses. Under these high-load conditions, the model incidentally produced remarks such as “its a more challenge,” “as best you can,” and even recall strategies such as “of the alphabet,” Such unprompted comments emerged only when list length exceeded the model’s comfortable span; with seven-item lists (the classic human limit; Miller, 1956), performance hit ceiling, and no commentary appeared. This mirrors long-standing findings that children’s private speech peaks when the cognitive demands of the task are high (Berk, 1992; Winsler et al., 2009). These preliminary results suggest that cognitive strain can elicit incidental private speech in an LLM like GPT-3.5-Turbo-instruct.

## 6 Conclusion

This study set out to determine whether a non-reasoning large language model (GPT-4o) can generate private speech and, if so, how its self-directed utterances align with those produced by humans. Our results show that the model reliably produced speech that was not socially addressed, satisfying the formal criteria for private speech; however, the proportions generated were not uniformly human-like. Rather than resembling the distribution reported by Winsler et al. (2003), our results differed largely. Conversely, GPT-4o was highly similar with Uçar and Sofu (2021), who used a similarly open-ended task. We stress that task nature differences play a role in comparison of our results versus human datasets. Furthermore, we show that with modifications in tasks (i.e., our exploratory serial recall task), incidental private speech may emerge under cognitive load, opening avenues for further inquiry into whether and how LLMs simulate the functional roles of private speech in humans, and potentially clarify computational consciousness.

## Data Availability

The datasets presented in this study can be found in online repositories. The names of the repository/repositories and accession number(s) can be found at: https://osf.io/t3us2/.
